# Replaying the tape of life in the twenty-first century

**DOI:** 10.1098/rsfs.2015.0057

**Published:** 2015-12-06

**Authors:** Virginie Orgogozo

**Affiliations:** CNRS, UMR7592, Institut Jacques Monod, Univ Paris Diderot, Sorbonne Paris Cité, 15 rue Hélène Brion, 75013 Paris, France

**Keywords:** evolution, convergence, tape of life, hotspot gene, contingency

## Abstract

Should the tape of life be replayed, would it produce similar living beings? A classical answer has long been ‘no’, but accumulating data are now challenging this view. Repeatability in experimental evolution, in phenotypic evolution of diverse species and in the genes underlying phenotypic evolution indicates that despite unpredictability at the level of basic evolutionary processes (such as apparition of mutations), a certain kind of predictability can emerge at higher levels over long time periods. For instance, a survey of the alleles described in the literature that cause non-deleterious phenotypic differences among animals, plants and yeasts indicates that similar phenotypes have often evolved in distinct taxa through independent mutations in the same genes. Does this mean that the range of possibilities for evolution is limited? Does this mean that we can predict the outcomes of a replayed tape of life? Imagining other possible paths for evolution runs into four important issues: (i) resolving the influence of contingency, (ii) imagining living organisms that are different from the ones we know, (iii) finding the relevant concepts for predicting evolution, and (iv) estimating the probability of occurrence for complex evolutionary events that occurred only once during the evolution of life on earth.

## 1. Introduction

At the end of the twentieth century, Gould [1, p. 48] popularized the thought experiment of ‘replaying life's tape’ and asserted that if we would press the rewind button—for example to go 600 million years back in time—and then run the tape again, the replay would be totally different. He viewed past and present organisms as a ‘subset of workable, but basically fortuitous, survivals among a much larger set that could have functioned just as well, but either never arose, or lost their opportunities, by historical happenstance’ [2, pp. 1160–1161]. From a wide diversity of body organizations that were present in the Cambrian only a few survived to present day, and with little change, as if, once evolved, animal body plans were constrained and could not freely change. Because no obvious supremacy was found among the multifarious Cambrian body organizations, Stephen Jay Gould suggested that mass extinctions were like lottery games. Had Cambrian conditions been slightly different, Pikaia would not have survived and the absence of vertebrates would have left room for other unfamiliar animals. Whether life's tape is rewound for a long or for a short time span matters for predicting the outcome: observing evolutionary trajectories that converge on a local fitness maximum over a short time period does not necessarily imply that they will reach the highest adaptive peak over a longer time period. The importance of stochastic events in the history of life is often illustrated by the asteroid impact that is thought to have led to the extinction of dinosaurs and the subsequent radiation of mammals 65 million years ago: had the meteorite not struck there would be no human to talk about it today.

As emphasized by Gould himself [1, p. 48], the question of whether life would replay the same is purely theoretical, because we cannot perform the experiment *per se* on all life forms on earth at once. In any case, most would agree that the tape of life would not generate exactly the same outcomes if initial conditions were slightly different. This paper deals here with a related but different question, the predictability of the outcomes: if life's tape is replayed can we make predictions about what to expect? What would happen if we rewind back to the Cambrian? And if we go back to before life itself appeared? Should we expect a DNA-based life? Trees with green leaves? Eye-like organs that are sensitive to light? The question of the level of predictability of evolutionary outcomes is, in my opinion, more interesting than Stephen Jay Gould's original question for at least two reasons. First, asking about the predictability rather than about the exact outcome of a replay does not polarize the debate between two extreme standpoints [3] and thus avoids the unnecessary clash between the Gouldian contingency adherents and the proponents of robust repeatability. Second, addressing this question of predictability can have important applied consequences on the design of efficient methods to search for life in other planets.

As of today, we can only have access to one run of the tape of life, the evolution of life on earth. So is it possible to tackle the question of the predictability of the outcomes of a replayed life's tape in a rigorous manner? This paper aims to show that it is. A reductionist approach is to fractionate the entire evolution of life on earth into partial bouts of evolution, either through time or through space (e.g. evolution of mammals in America and in Australia), to identify elements that occurred in multiple instances during life evolution, and to look for repetitive patterns that can be framed in terms of causes and effects. To become more amenable to experimentation, Gould's original question is thus reformulated into several questions [4]. If independent lineages are subjected to the same environmental conditions, how often will they evolve the same phenotypes? And what do we mean by ‘same’? Does independent evolution of the same phenotypic state often involve the same kinds of mutations? From convergence towards local adaptive peaks during short time frames can we infer that evolution will also converge towards the highest peaks over longer time periods? In the past 10 years have come forth a series of books and websites [5–9] that suggest that the evolutionary pathways available to life are not endless, but might be quite limited in number and possibly predictable. The major argument put forth in favour of predictability is that during past evolution similar traits have evolved independently multiple times in diverse taxonomic groups.

Today, three types of data in biology reveal a certain level of repeatability in evolution: experimental evolution, studies of convergent evolution across various species and evolutionary genetics. After reviewing these three kinds of experimental evidence, this article reflects on the notion of predictability. In evolutionary biology, predictions are not necessarily based on complex mechanistic models; they can simply derive from the observation of repeated evolution and from the identification of the conditions that lead to repeated outcomes. This paper examines whether current data imply (i) that the range of possibilities for evolution is limited and (ii) that predictions can be made about the outcomes of a replayed tape of life. I identify four important issues that need to be addressed for trying to unravel the outcomes of a rewinded tape of life.

Viewing evolution as a path in which both time and successive states are represented within a three-dimensional space is intuitive and widespread [10,11]. Our language is full of space–time metaphors [12], such as ‘holidays are *approaching*’, and these often help to grasp the notion of time. This paper is no exception and makes ample use of the metaphor of ‘evolutionary paths', although it is wise to remember that any metaphor is likely to carry negative analogies, i.e. features that are not shared between the source of the metaphor and its target [13].

## 2. Repeatability in experimental evolution: phenotypic and genetic paths are limited

A typical experiment for testing the repeatability of evolution is to set up several populations of individuals in the same experimental conditions and let them evolve independently. If the populations evolve in a similar manner, then the number of evolutionary paths is considered limited, and evolution is concluded to be predictable. If no pattern is observed, then no prediction can be made, besides maybe that populations will adapt to the experimental conditions. For example, 115 replicate populations of an *Escherichia coli* strain initially adapted to 37°C were grown at 42.2°C for 2000 generations [14]. At the end of the experiment, a single clone of each population had its genome sequenced and its fitness estimated. All lines survived better and produced more progeny at 42.2°C than their ancestor. More than 1000 mutations were identified, and most seemed to affect two main pathways, involving either the RNA polymerase complex or the termination factor rho. In another experiment, eight independent populations of yeasts were grown in a medium with low sulfate [15]. After about 200 generations, all populations exhibited a 50% increase in fitness or more. The sequencing of two clones per populations revealed an amplification of the gene *SUL1*, which encodes a high affinity sulfate transporter, in 15 of 16 clones and several coding changes in *SUL1* in the remaining clone. The number of *SUL1* copies varied from two to 16 and the amplified genomic region ranged from 2.5 to over 40 kb, with different breakpoints in each clone. No such *SUL1* mutations were detected in control conditions with normal sulfate concentrations. In this case, adaptation to low sulfate involved mutations in the *SUL1* gene in all clones.

The two above-described experiments used individuals that had all the same genome sequence at the beginning of the selection regime, so that evolutionary changes were forced to occur through mutations appearing de novo during the experiment. Alternatively, experimental evolution can be performed on different replicates of an initial population that harbours standing genetic variation, or on populations with distinct past histories. Recent experiments with yeasts and *Drosophila* flies indicate that evolution resulting from standing variation is more repeatable than evolution resulting from de novo mutations [16–18]. In a very recent laboratory experiment, distinct populations of *Drosophila subobscura* originating from three different latitudes were observed to evolve independently towards the same body size, same fecundity rate and same starvation resistance level in only 22 generations [19]. This suggests that laboratory selection can quickly erase differences between populations.

**Figure 1.**
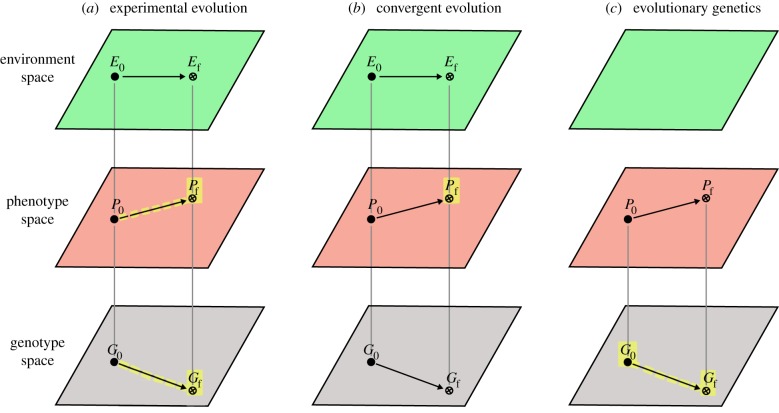
Three kinds of predictability. The environment space corresponds to all possible environments, the phenotype space to all possible phenotypes and the genotype space to all possible genotypes. The black point represents the initial state at *t* = 0 (*E*_0_, initial environment, *P*_0_, initial phenotype, *G*_0_, initial genotype). The crossed point corresponds to the final state (*E*_f_, final environment, *P*_f_, final phenotype, *G*_f_, final genotype). For simplification, a point in genotype and phenotype space corresponds here to one individual. Vertical bars indicate associations between genotypes, phenotypes and environments. Predictions are highlighted in yellow. (*a*) Experimental evolution studies suggest that if *P*_0_, *G*_0_ and *E*_f_ are known, then for certain cases we can predict *P*_f_ and *G*_f_. (*b*) Studies of convergent evolution suggest that if we know E_f_ then for certain cases we can predict *P*_f_. (*c*) Evolutionary genetics suggests that if we know *P*_0_ and *P*_f_, then for certain cases we can predict *G*_0_ and *G*_f_.

Several evolution experiments such as the ones described above have found a certain degree of repeatability, with multiple instances of adaptation occurring through the increase in frequency of the same segregating alleles or through independent de novo mutations either at the same nucleotide position, in the same gene or affecting the same gene expression profile or the same pathway [20] (table 1). From these results, one might be tempted to conclude that if independent lineages are subjected to the same environmental conditions then they will often evolve the same phenotypic traits through a limited set of possible genetic changes (figure 1*a*). However, as we will see in the next paragraphs, several unknowns remain before we can draw such conclusion.

**Table 1.** Various levels of predictability.

level of convergence	phenotype	organism	references
*nucleotide*			
coding mutations in several genes	rate of proliferation in a novel host and at higher temperature	X174 virus	[21]
Gly119Ser in *ace-1*	organophosphate resistance	seven insect species	[22,23]
*gene*			
*pykF* and *nadR*	rate of proliferation in a glucose-limited medium	*Escherichia coli*	[24]
*yellow*	wing spot	*Drosophila tristis* and *Drosophila biarmipes*	[25]
*protein accumulation profile*			
proteins regulated by guanosine tetraphosphate	rate of proliferation in a glucose-limited medium	*E. coli*	[26]
*molecular pathway*			
RNA polymerase complex	rate of proliferation at high temperature	*E. coli*	[14]
*organ*			
compound eye	sensitivity to light	certain annelids, arrow worms, insects	[7]
*behaviour and organs*			
no sound production and flat wings	no sound production, protection against parasitoids	criquets	[27]
feeding behaviour and habitat adaptations	ecology and body morphology	lizards	[28]

Most experimental evolution experiments have been performed on microorganisms (such as bacteria, yeasts or malaria parasites) because they are particularly well suited to such experimentation [29]. The largest and most famous laboratory-controlled experiment, which has been running for more than 25 years and is still ongoing, has been using *E. coli* bacteria [24,26,30]. Experimental evolution involving sexual reproduction and multicellular organisms with long life cycles has proved difficult for practical reasons. Whether results obtained with microorganisms can be generalized to macroorganisms remains unclear [18]. Furthermore, with experimental evolution, it is only practical to study a small number of generations, and in environmental conditions that are often too simplistic compared with the real conditions of life, where multiple species interact in complex and changing environments. Even though recent experimental evolution studies aim at reconstructing evolutionary steps that resemble major transitions, such as the evolution of multicellularity [31,32], other complex evolutionary changes, such as the endosymbiosis event that led to the evolution of mitochondria, seem to be inaccessible to experimental evolution because they would probably require several thousands or millions of years of artificial evolution.

In conclusion, experimental evolution has uncovered cases where independent lineages subjected to the same environmental conditions have evolved the same phenotypic traits via a limited set of possible mutations. Whether this observation would hold true in all taxa remains uncertain.

## 3. Repeatability in phenotypic evolution of distinct species: phenotypic paths are limited

Convergent evolution occurs when several lineages independently evolve similar or identical phenotypic traits. Amazing examples of convergent evolution have been compiled by various authors in recent years [5–8]. For example, animals that swim in the dense medium of water have all evolved streamlined, fusiform morphologies or eel-shaped bodies. The fusiform body of the extinct Mezosoic marine reptile *Ichthyosaurus*, dolphins and many fish species is a spectacular case of convergent evolution [7]. At least 49 independent lineages of animals have evolved light-detection organs that contain aggregates of photoreceptor cells [7]. Vision organs can be classified into six types: eyespots (photoreceptor cells aggregated into spot regions), ocellar pits (photoreceptor cells located in an open pit, connected to an optic nerve), ocellar cups (photoreceptor cells located in a partially enclosed cup-shaped structure, connected to an optic nerve), simple eyes (globular eye with pinhole opening or closed opening), compound eyes (complex eye with multiple lenses) and camera eyes (complex eye with a single lens). For each type, at least five instances of convergent evolution have been reported [7]. Another striking example is the convergence between the marsupials of Australia and the placental mammals of the rest of the world. Thirteen ecological analogues can be found, corresponding to the great cat, the small cat, the wolf, the wolverine, the anteater, the flying squirrel, etc.

The ever-growing compilation of cases of phenotypic convergence [6] indicates that the evolutionary process repeats itself at multiple levels, from molecules to ecosystems. Such observations suggest that we can make predictions about the evolution of phenotypes in the future, past and present: given a set of environmental conditions, then certain kinds of phenotypes are expected (figure 1*b*). For example, George McGhee predicted that ‘if any large, fast-swimming organisms exist in the oceans of Jupiter’s moon Europa, swimming under the perpetual ice that covers their world, […] they will have streamlined, fusiform bodies; that is, they will look very similar to a porpoise, an ichthyosaur, a swordfish, or a shark’ [7, p. 272].

A problem with compilations of convergent evolution that happened during our past evolution is that it can be difficult to identify the environmental conditions associated with the evolution of convergent traits. In general, biologists cherry pick examples of phenotypic convergence and then try to identify the environmental feature in common to explain it. Environmental conditions can only be guessed, and our intuitions of the underlying selective pressures may sometimes be incorrect [33]. For example, white body colour has evolved independently in numerous animal species that live in snowy habitats. It seems obvious that this convergent evolution pattern is due to selection for camouflage in both prey and predator species. An exception to this rule is the snow flea, a 1.5 mm long springtail that can be readily observed on the surface of snow, because its dark colour contrasts sharply with the white background. Here, one has to invoke other causes to explain its black body, such as selection for retention of sunlight energy and absence of predators. Furthermore, the large diversity of species that is usually found in a given place suggests that many phenotypic solutions exist to a particular environment.

In conclusion, compilations of convergent evolution indicate that if the set of environmental conditions faced by living beings is known, then we may somehow, in some cases, predict the outcome in terms of phenotypic properties.

## 4. Repeatability in the genes underlying phenotypic evolution: genetic paths are limited

Owing to tremendous progress in sequencing technologies, the genes and the mutations responsible for evolutionary changes between species or populations are now being identified at an increasing pace. As of today, we enjoy a catalogue of more than 1000 mutations and genes responsible for independent non-deleterious difference in morphology, physiology and behaviour in animals, plants and yeasts [34]. These ‘loci of evolution’ [35,36] have been identified through two major approaches, either based on an *a priori* assumption of gene function (candidate gene approach), or based on correlations between segregating genomic regions and phenotypes within populations (genomic mapping). Among the catalogue of loci of evolution, 111 genes, named ‘hotspot genes', are found repeatedly and are responsible for more than half (611/1008) of the cases where a gene has been associated with a phenotypic change [36]. For example, the *oca2* gene has been associated with loss of body pigmentation in both cave fishes and humans via distinct mutations [37]. In butterflies, *WntA* has been associated with evolutionary variation in wing colour pattern in at least five species, some of them having diverged from each other more than 65 millions of years ago [38–40]. The ability to synthesize carotenoid pigments has evolved independently in at least three taxa, pea aphids, gall midges and spider mites [41–43], always through lateral transfer from distinct fungi donors of a homologous genomic region containing three enzyme genes. The redundancy in the catalogue is so high that we may feel tempted to predict, for a given phenotypic variation, the underlying genetic changes, at least at the gene level (figure 1*c*). As a matter of fact, the field of evolutionary genetics is starting to fill up with stories about researchers predicting candidate genes, which proved correct after several years of intense mapping work (A. Martin, F. Roux, M. Tsiantis 2013, 2014, personal communications).

It is important to note that the catalogue of loci of evolution contains multiple biases (reviewed in references [35,36]). When a gene has already been found to be responsible for an evolutionary difference, researchers who investigate the genetic basis of a similar phenotype in another taxon tend to study preferentially the gene that was previously identified rather than other genes. Furthermore, with the candidate gene approach genes that are already known from developmental biology or physiology studies are more likely to be identified than unstudied genes. This means that within the catalogue hotspot genes are likely to be over-represented. In addition, cases where the candidate gene approach failed and the tested gene was found not to be involved are not compiled in the catalogue. As a consequence, the catalogue cannot be used to derive the likelihood of certain genetic changes given a particular phenotypic change. However, a few studies have tried to quantify repeatability at the genetic level. To estimate the contribution of the hotspot gene *FRIGIDA* to natural variation in flowering time in *Arabidopsis thaliana*, *FRIGIDA* coding sequences and flowering time were examined in 192 worldwide populations [44]. The authors found that approximately 70% of flowering time variation could be accounted for by allelic variation of FRIGIDA. In sticklebacks, a deletion in the *Pitx1* gene is associated with pelvic loss [45]. A survey of 13 pelvic-reduced populations from disparate geographical locations identified nine independent deletions with distinct breakpoints, all affecting a 488-bp region that drives *Pitx1* expression in the developing pelvis. Still, four populations had reduced pelvis but no deletion in the region of interest of *Pitx1*.

The catalogue of loci of evolution is also biased towards species pairs that can give hybrid progeny, towards species that can be raised in great numbers in the laboratory [46] and towards large-effect loci because they are the easiest to identify [47]. Whether trends emerging from the catalogue would also apply to other types of loci and species is unclear. In any case, compilation of current data on the genetic basis of evolutionary change suggests that for certain phenotypic changes we can predict the underlying genetic variation with higher confidence than what most biologists had thought 20 years ago [48].

## 5. Three types of predictions

Making a prediction in physics usually means inferring the final state(s) based on information about the initial conditions and on a model. Here, the predictions that can be made about life evolution based on the observation of repeatability are of a different kind. They rely on knowledge of certain parameters of the final state (final environment or final phenotype) and they do not require a good understanding of why repeatability exists. While certain authors have been attempting to develop evolutionary models based on gene networks that can help to predict the genes underlying phenotypic evolution [35,49–51] and others have guessed which human influenza virus strains will circulate next winter based on genealogical trees [52], most predictions in evolutionary biology are simply based on the identification of the conditions that are thought to cause repeated outcomes. Predictions in evolutionary biology can be represented graphically, where evolution is shown as a path within three spaces, the environment, phenotype and genotype spaces (figure 1*a–c*). Experimental evolution studies suggest that if certain parameters of the initial states and the final environment are known, then for certain cases we can predict the final phenotype and sometimes the final genotype (figure 1*a*). Studies of convergent evolution across taxa suggest that if we know the final environment then for certain cases we can predict the final phenotype (figure 1*b*). Evolutionary genetics suggests that if we know the difference in phenotype between the initial and final state, then for certain cases we can predict the genotype difference (figure 1*c*). Because adaptive evolution of phenotypes and genotypes is considered to occur once organisms are allowed to adapt in the environmental conditions, cases 1*a* and 1*b* are close to typical predictions about the future. In contrast, case 1*c* is a prediction of events occurring rather simultaneously at the genotype and phenotype levels: what is predicted is the genetic change underlying a given phenotypic change [48]. Furthermore, the three acts of predictions presented here can apply to evolutionary changes that occurred in the past, that are occurring today or that might occur in the future. We note however that despite its explanatory power the graphical representation of figure 1*a–c* has several flaws. First, it artificially dissociates genes from environment, whereas the effects of genes and environment on phenotypes cannot be isolated [48,53]. Second, environmental conditions and adaptive pressures are often dependent on the organisms themselves: the fitness of an individual or of a genotype may depend upon its frequency in the population, or upon properties of other cohabiting species.

In summary, the observation of repeatability in three contexts (in experimental evolution, in convergent phenotypic evolution across taxa and in the genes underlying phenotypic evolution) suggests that predictions are possible about the phenotypic and genotypic traits of evolving organisms.

## 6. What is the null hypothesis? Thinking about other possible paths

Many researchers have expressed surprise when they discovered that the same genes had mutated independently over and over to cause repeated phenotypic evolution. According to their intuition, multiple genes in a genome could have mutated and led to a given phenotypic change. Furthermore, the range of possible phenotypes that are adaptations to a given environment appears to be so large that it seems improbable that similarities could have arisen by chance. A conclusion emerging from the above-presented data is that evolution is more limited and more repeatable than expected. But actually, what did we expect? To conclude that the paths of evolution are limited, we need a null hypothesis of the possible paths and outcomes in the phenotype space, and possibly in the genotype space. If we can show that the observed evolutionary outcomes represent only a subset of all possibilities, then we can conclude that evolution is more limited than expected. For example, a null hypothesis for the mutations responsible for evolution could be that each nucleotide site within a genome has an equal probability of mutating. In many instances, estimating the probability of occurrence of a given event relies on assumptions based on common sense. Because these assumptions cannot be formally demonstrated, distinct hypotheses are usually possible, and each one can thus lead to the calculation of a different value of probability of occurrence of an event of interest [54, ch. XI.III]. Therefore, when we want to address the question of predictability in evolution, it is important to reflect on the null hypothesis.

What is the null hypothesis of possible paths and outcomes in the phenotype space and in the genotype space? Imagining possible states is simpler for the genotype space than for the phenotype space. One possibility is to consider that it corresponds to all possible strings of nucleotides, with each nucleotide having an equal probability of occurrence. But this becomes quickly unmanageable. For just a small DNA molecule of 10 base pairs, more than one million sequences are possible. Besides viruses perhaps, it is thus impossible to test for all genetic paths. The largest study so far, a tour de force, examined nearly all possible 24-nucleotide RNA molecules (more than 10^14^ molecules) for binding to GTP agarose resin [55]. Another strategy is to examine multiple possible paths starting from the ancestral state. In cases where only the mutations with largest effects are included in the null model, the resulting model is biased [4]. A better solution is to start from a library of random mutants obtained from an initial sequence. Mutation accumulation lines, i.e. inbred lines in which mutations accumulate, have been used to estimate the rates and properties of new spontaneous mutations [56]. Unfortunately, it is often impossible to obtain all possible mutations from an initial sequence because they are too many. Practically, a smaller sample of mutations is studied. To make it as representative as possible, the sample is obtained through a random process. In a recent yeast study [51], 236 of all 241 possible G : C → A : T transitions were individually introduced in an initial DNA sequence [57]. Researchers focused on G : C → A : T transitions because they were the most common type of single nucleotide polymorphism observed among laboratory and natural strains of *S. cerevisiae*. Overall, no significant difference in gene expression level was found between the effects of G : C → A : T and other types of polymorphisms previously studied. An alternative strategy is to focus only on the mutations that did occur from the ancestral state to the derived state and examine all possible orders of these mutations [58,59]. Still, the number of possible states increases exponentially with the number of evolutionary steps. So far, systematic studies have shuffled between three and nine mutations, either within a single gene or across genes [4,20,60]. Another possibility is to use the distribution of laboratory-induced mutations as a null hypothesis. For example, more than 100 genes in the *D. melanogaster* genome have been found through mutagenesis screens to affect hair pattern [35], whereas evolution of hair pattern in two *Drosophila* species has been shown to involve at least 12 *cis*-regulatory mutations in a single gene, *shavenbaby* [61].

**Figure 2.**
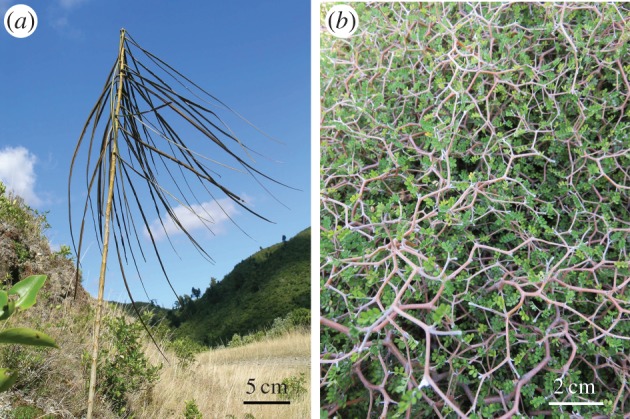
(*a*) Picture of a juvenile lancewood *Pseudopanax crassifolius* (credit: Leon Perrie, Wellington). The ratio of leaf length over central stem length is much higher than in other plants. (*b*) Picture of a shrub of *Sophora prostata*. (credit: Virginie Orgogozo, Paris). Except in New Zealand, shrubs display no such intricate mesh of stems with small leaves.

Imagining the null hypothesis of all possible phenotypes and their probability of occurrence can be done in various ways. First, some researchers have examined all possible DNA sequences to infer all possible phenotypes, but such studies have only considered extremely simple phenotypes that are directly linked to the activity of a single gene [20], such as GFP expression level [57], enzyme activity [62], binding affinity [55,59] or antibiotic resistance [58]. Second, characters that are found in distinct organisms can be combined within an imaginary, chimeric organism [63,64]. Third, one can extract parameters from physics or biology (gravitational constant, number of arms, index of butterfly wing colour pattern, rate of whorl expansion for a coiled shell) and let them vary within and outside the range of observed values [65]. Fourth, one can draw analogies with non-living objects, for example when imagining animals that would move using rotating wheel-like organs [66]. Whatever we do, it appears that we always have to rely on our own world to think of all possible phenotypes [67, p. xv]. Even a silicium-based world is imagined in reference to our carbon-based life [9]. The way we can imagine other possible worlds might therefore bias our thoughts towards certain outcomes. As a matter of fact, the incredible shapes of certain New Zealand plants, such as the juvenile lancewood or shrubs of divaricating growth (figure 2*a,b*), would probably not have been imagined if unseen. Maybe there is a better way to perceive light than with eye-like organs but we cannot think of it.

Traditionally, the aim of evolutionary biology has been to unfold and understand our past evolution, that is *how and why it happened this way* [68]. The ‘why’ question of biology traditionally meant why this change occurred rather than not (because of selection, drift, migration, etc.). But the ‘why’ question in biology can also ask *why this change occurred rather than another change*. To tackle the latter, several current research fields in biology are not only dissecting the path taken during past evolution, but also investigating other paths that were not taken and comparing them with the one that occurred.

Overall, accumulated data suggest that the path taken by evolution displays particular properties that differ from those of paths not taken and that the end-result corresponds to the optimization of certain parameters. On the genetic side, the path taken during evolution seems to be the one that involves no or few steps that decrease fitness, few steps that do not change fitness (neutral facilitating mutations) and mostly steps that increase fitness. Furthermore, the mutations involved in natural evolution appear to be the ones with fewer pleiotropic effects [35] and with no increase in gene expression noise [57] compared with random mutations. On the phenotypic side, living and extinct organisms are often found to harbour a limited range of values for particular parameters describing their phenotypes. For example, in aquatic animals using long fins to swim, the length of an undulation along the animal's fin divided by the mean amplitude of undulations along fin length is consistently about 20 [69]. Computer simulations and robotic fins show that among physically possible scenarios a ratio of 20 maximizes both swimming speed and the force generated by the body. Similarly, physical considerations on a broader scale suggest that there might be only a handful number of ways to arrange an image-forming eye of substantially high acuity owing to the laws of optics and the properties of light [70]. Using autonomous three-dimensional virtual creatures in an unlimited hyperspace, Karl Sims’ computer program evolved creatures that display various modes of locomotion, with water- and land-based movements such as swimming, jumping and walking [71]. A few of his evolved modes of locomotion are unknown from actual organisms, suggesting that certain possible evolutionary paths have not (yet?) been taken during life evolution on earth. Importantly, the number of evolved modes of locomotion was found to be limited.

Optima based on physical considerations are also sometimes proven wrong by biology studies. For example, optical considerations for a wide aperture pinhole camera predicted that the resolution of the infrared detection system in pit vipers should be very low, but experimental studies showed that snakes can orient to heat with an angle accuracy of 5°. Subsequent modelling showed that a simple neural network can allow the formation of a clear neuronal image of the spatial heat distribution despite a blurred heat distribution image on the pit membrane [72]. The complexity of life sometimes makes it difficult to elaborate relevant physical models.

In conclusion, efforts are being made on delineating the null hypothesis of all possible paths in the genotype and phenotype spaces. On the phenotypic level, physical considerations and computer simulations suggest that certain conditions call for the same optimal phenotypes and that these are observed in living or extinct organisms. On the genetic level, the mutations involved in natural evolution appear to form a particular subset of all possible mutations: they are the ones with little maladaptive effects, no increase in gene expression noise and very specific (non-pleiotropic) effects.

## 7. How influential is the initial state?

The three kinds of repeatability described above—in experimental evolution, in phenotypic evolution of distinct species and in the genes underlying past evolutionary changes—show that we can make predictions about evolutionary outcomes if we know about the initial and final states of the environment and/or of the phenotype (figure 1*a–c*). But a crucial question remains: what is the range of initial conditions that would lead to the predicted outcome? Would we end up with the same outcome if initial states were different? For example, as far as we know, all living organisms carry DNA or RNA. So how can we be sure that other possible kinds of living organisms that would be devoid of DNA or RNA would follow the same rules if we have no data on such organisms? Phylogenetic studies indicate that all the species living on earth share the same origin. Therefore, no pair of evolutionary events can be considered as totally independent of each other. Convergent evolution of the ability to synthesize carotenoids in two insect species and one mite species [41–43] has been possible because these three species exhibit a similar metabolism and possess the relevant substrate molecules for carotenoid synthesis. Had the metabolic substrate not existed in these species, genetic changes affecting other genomic regions would have been involved in their acquiring a carotenoid synthesis pathway.

Several observations suggest that the prior state can influence the outcome of subsequent evolution. In an evolution experiment of 12 populations of *E. coli*, aerobic citrate utilization arose after about 31 000 generations in only one population [30]. The other populations never evolved citrate use, but clones isolated from the evolved population at various time points before the appearance of citrate utilization had high probability to evolve citrate use. This observation and others indicated that evolution of citrate use was contingent upon the prior appearance of several potentiating mutations which have no apparent fitness effect alone [30]. In another experimental setting, two phage populations grown in the same conditions repeatedly evolved towards distinct outcomes and at distinct rates [73]. Several studies suggest that when the sign of a mutation's fitness effect depends on its genetic background (sign epistasis), the initial mutation can strongly constrain the paths of evolution [20,60]. For example, *in vitro* evolution of the antibiotic resistance enzyme TEM-1 β-lactamase mostly occurs through three ‘good’ mutations in a fixed order [74]. But a few deviating lines evolve comparatively lower resistance, and this is due to their accumulation of initial mutations that prevent the three ‘good’ mutations to have a positive effect on resistance. The fate of an adaptive mutation can also be influenced by the fortuitous presence of other adaptive mutations in other individuals of the evolving population [75]. In mosquitoes, high level of organophosphate resistance is conferred by a single glycine to serine substitution at position 119 of the acetylcholinesterase protein [22]. Resistance was observed in five mosquito species but not in 12 others. The species which evolved resistance required only one nucleotide change (glycine 119 encoded by GGC), whereas the others would have needed two nucleotide changes (glycine 119 encoded by GGA or GGG) to evolve resistance [22]. Here, the initial DNA sequence determines whether resistance will evolve or not. Organophosphate resistance has also evolved in mosquitoes through esterase gene amplification at least seven times independently [76]. Interestingly, organophosphate resistance in Brachycera flies has never been associated with *esterase* gene amplification, even though the gene is present and amino acid changes in the esterase have been shown to cause resistance in the fly *Lucilia cuprina* [77]. Here again, it seems that the initial genome (whether fly or mosquito) predetermines which mutations will confer resistance. A meta-analysis of approximately 25 cases suggests that independent evolution of the same phenotype is more likely to involve mutations in the same orthologous genes when they are closely related species than when they are distantly related species [78].

In brief, experimental data show that initial conditions constrain evolutionary paths and that changes in the initial conditions can affect the probability of evolutionary outcomes. This observation can be interpreted in two alternative views [3]: (i) the initial state was the only possibility, and then paths are limited and thus predictable or (ii) the initial state was one out of many other likely states, and then evolution is not predictable but contingent on previous events that are unpredictable. Even if (ii) is true, it can be argued that if more time is allowed, then a path that was not available for short-term evolution may become accessible, and may lead to predictable outcomes over longer time periods. This point raises the issue of whether high fitness peaks are always accessible by a random walk in total space [79,80]. Maybe the *E. coli* lineages that did not evolve citrate use would do if they were allotted more time. An area of the phenotype space that is not accessible locally might become accessible through a convoluted path. Interestingly, the proportion of *cis*-regulatory mutations responsible for morphological changes (relative to coding mutations) is higher for interspecific changes than for intraspecific changes in animals and plants [35,46]. This is consistent with short-term evolution involving strong and pleiotropic mutations which are not fixed within species, whereas over longer time periods, these mutations are replaced by rarer ones that have more subtle and specific effects [81]. Events that are unlikely within short evolutionary timescales may become likely over longer time frames.

Overall, it is unknown whether highest peaks are reachable from any initial state, that is whether evolution can always bypass developmental constraints, pleiotropy, epistasis, genetic drift or cases where optimality in one trait is associated with suboptimality in another trait. In vertebrate eyes, the passage of retinal axons across the retina creates a blind spot. Evolution of an upside-down retina, so that the vertebrate eye would resemble a cephalopod eye, would have eliminated the blind spot, but this has never been observed. Instead, neuronal mechanisms have evolved for vertebrates to acquire a complete field of vision despite their blind spot. Here, it seems that the vision defects associated with the blind spot cannot be readily overcome through changes in retinal morphology, but through changes in downstream neural networks.

## 8. Finding the relevant level for predicting evolution

Even though many stochastic processes lie at the heart of the evolutionary process (table 2), the three kinds of data summarized above show that partial bouts of evolution can be somehow predictable: certain environments can be predicted to be associated with certain phenotypes, and certain phenotypic changes with particular genetic changes (figure 1*a,b*). But how can we reconcile this predictability with the inherent stochasticity of the evolutionary process? An illuminating analogy is the behaviour of an ideal gas in a container. At the microscopic level, the position, mass and velocity of each point particle are unpredictable. However, at the macroscopic level, other characteristics such as pressure, temperature, number of moles are predictable. Even though the behaviour of single particles is unpredictable, their average and standard deviation is. Similarly, even though mutations arise in an unpredictable manner, predictability can emerge over longer timeframes at the level of the mutations underlying phenotypic evolution and at higher levels owing to selection.

**Table 2.** List of unpredictable phenomena that are part of the evolutionary process. These events are said to occur ‘by chance’, i.e. they are not explained by our current theories, they cannot be predicted to occur or there is no finality/purpose in the event itself [82].

error in DNA replication
cosmic rays causing mutations
position of the mutations across the genome (mutation rate vary with position along genomes, many sites within a genome are expected to mutate with a non-null probability)
chromosome segregation during meiosis
assortative mating between individuals
gamete competition during fecundation
genetic linkage between genetic loci
unpredictable environmental changes such as meteorite impact

Most would agree that if a life form evolves, then it is predicted to process chemicals, to replicate, and to allow a kind of heritable variation, so that evolution through natural selection can take place. This is actually one definition of life itself [9]. A slightly more advanced prediction is that life forms should be carbon based [9]. How far can we go in our predictions? Can we predict like Simon Conway Morris that there should be trees and that these trees should be green because there is no better molecule than chlorophyll to convert light energy into a redox reaction [5]?

As emphasized in the Introduction, the crucial question is not whether evolution is predictable but at which level predictions can be made if life's tape is replayed. There are more than one billion of possible 30-nucleotide RNA molecules composed of A and U nucleotides, but computer analysis shows that they fold into only about 1000 shapes [83]. Can we find RNA shape-like concepts for predicting evolution? Streamlined fusiform bodies, tree-like shape, wings are such general concepts that might apply to any fast-swimming, land photosynthetic and flying organisms, respectively. The tricky point is to find concepts that are large enough, so that they encapsulate as many cases as possible, and that are precise enough, so that they contain information. Predictability can exist at the molecular level, at the nucleotide level, at the gene level, at the pathway level or at various levels regarding the phenotype (table 1). The level of predictability probably depends on the context (time, environment, phenotype, genotype, etc.). For example, *E. coli* resistance to trimethoprim localized only to the *DHFR* gene, whereas resistance to chloramphenicol and doxycycline involved mutations in about a dozen genes involved in translation, transcription and transport [84].

## 9. Extrapolating from short bouts of evolution to the entire span of life evolution

One conclusion emerging from experimental evolution and genetic analysis of past evolution is that evolution seems to follow a limited set of genetic and phenotypic paths at a given time point and space point during evolution. Nevertheless, this limited set still contains several paths. For example, experimental evolution of wrinkly spreaders in *Pseudomonas fluorescens* occurs exclusively through mutations in three pathways in the laboratory (*n* = 26 replicates), but elimination of these pathways uncovered 13 other mutational pathways to wrinkly spreaders [85]. When we want to address the predictability of evolution, we want to know whether there is a single path, and in cases where there are multiple paths, whether these different paths can be grouped together because they exhibit a shared characteristic feature. What matters is not whether the number of possible evolutionary paths is limited but its order of magnitude. If there are multiple paths on a short time scale, then there will be a multitude of possible paths over a long time period. Furthermore, even though the number of possible paths is restricted compared with all imaginary paths, the entire space of possibilities might still be infinite.

A common argument put forth against the predictability of evolution is that certain events occurred only once, or a small number of times, during the evolution of life (evolution of the genetic code, of mitochondria, of neurons, of neural crest cells, of an intelligence that allow us to reflect on our own evolutionary origin [86]). Would such events appear again if life's tape is replayed? How can we infer that various starting conditions would inevitably trigger these rare evolutionary events? How come marsupials did not evolve the ability to fly while placental mammals (bats) did? If sapient beings are a predictable outcome of evolution, then why did they arise only once, in primates in Africa, and not from mammals in America? Is it because such phenotypes, once they appeared, colonized all available niches and thus prevented other organisms from taking the long route for evolving the same phenotype? Or is it because such events are highly unlikely and thus unpredictable? A pressing issue for assessing the predictability of life's tape is to estimate the likelihood of important events that occurred only once or very few times during the evolution of life on earth. Artificial evolution of complex events in a laboratory setting from simple initial conditions such as evolution of DNA, genetic code or nervous cells seems like an unreachable endeavour, but recent studies showed that previously unthinkable feats might not be so difficult to achieve in the laboratory after all (for example evolution of multicellularity [31,32] and evolution of ribonucleotides [87]).

The data reviewed above show that certain small portions of evolution can be predicted. In each case, predictions are made given certain conditions. While certain researchers investigate whether life is likely to evolve DNA, cells or lipid cell membranes in diverse environmental conditions, others investigate whether cells with DNA are bound to evolve transcription factors, and yet others whether unicellular organisms with DNA are likely to evolve into multicellular organisms with a circulatory system. Then, to derive general trends about the entire evolution of life, we need to know the probabilities of occurrence of all these portions of life evolution and to aggregate them all. Even though one can rejoice that the important question of the predictability of life evolution has now become amenable to experimental analysis, its field of investigation is tremendous.

## 10. Conclusion

The question of whether outcomes of a replayed life's tape are predictable is now being addressed with an experimental approach, through a series of investigations dealing with smaller bouts of evolution. While it is too early to derive any definite conclusion, recent observations suggest that there are predictable portions within life's tape and that evolution might not be as unpredictable as once thought 25 years ago, when Stephen Jay Gould formulated his original question.
